# A mental health program for infertile couples undergoing oocyte donation: protocol for a mixed methods study

**DOI:** 10.1186/s12978-020-0865-8

**Published:** 2020-01-22

**Authors:** Shohre Ghelich-Khani, Ashraf Kazemi, Malek Fereidooni-Moghadam, Mousa Alavi

**Affiliations:** 1 School of Nursing and Midwifery, Isfahan University of Medical Sciences, Isfahan, Iran; 2 Nursing and Midwifery Care Research Center, School of Nursing and Midwifery, Isfahan University of Medical Sciences, Isfahan, Iran

**Keywords:** Oocyte donation, Infertility, Mental health, Protocol study

## Abstract

**Background:**

The psychological consequences of infertility in couples undergoing oocyte donation differ culturally, racially, religiously, and legally from other infertile couples undergoing assisted reproductive treatments. Therefore, the inclusion of a mental health program in assisted reproductive services is essential for these couples. As such, the aim of this study is to develop a program for improving the mental health of these couples.

**Methods:**

This study is designed using an exploratory mixed method and the program based on Talbot and Verrinder model. Different steps of this research include determination of a specific topic for planning (needs assessment), initial design of the program, finalization of the program (using the views of experts in this area), implementation of the program, monitoring of the implementation of the program and evaluation of the program. To perform the first step of Talbot’s program, the first phase of the study will be conducted. At first, through a qualitative study, the items of the questionnaire are designed and then its psychometric steps will be performed by a cross-sectional study. In the second and third steps, the classic Delphi technique will be used in four-round for initiation and finalization of the program, and the second phase will be completed. The fourth, fifth and sixth steps of the program including implementation, monitoring of the implementation and evaluation of the program in the future will be performed.

**Discussion:**

Designing an appropriate program based on the documentations of the qualitative study and evidence can improve the mental health and quality of life of the couples undergoing oocyte donation. The program, based on the measurement of needs, will be implemented using a tool designed specifically for the target population and can be useful in the processes of treatment, education, policymaking and legislation as well as research.

## Plain English summary

Studies have shown that among needs, emotional and psychological needs are the most important ones. According to the evidence, infertility-related psychological problems, including stress and anxiety, can be a contributing factor to the exacerbation of infertility and failure in the process of its treatment. The psychological consequences of infertility in couples undergoing oocyte donation differ culturally, racially, religiously, and legally from other infertile couples undergoing assisted reproductive treatments. Therefore, the inclusion of a mental health program in assisted reproductive services is essential for these couples. As such, the aim of this study is to develop a program for improving the mental health of these couple. This study is designed using an exploratory mixed method based on Talbot and Verrinder model. Different steps of this research include determination of a specific topic for planning, initial design of the program, finalization of the program (using the views of experts in this area), implementation of the program, monitoring of the implementation of the program and evaluation of the program. To perform the first step of Talbot’s program, the first phase of the study will be conducted. At first, through a qualitative study, the items of the questionnaire are designed and then its psychometric steps will be performed by a cross-sectional study. In the second and third steps, the classic Delphi technique will be used in four-round for initiation and finalization of the program, and the second phase will be completed. The fourth fifth and sixth steps of the program including implementation, monitoring of the implementation and evaluation of the program in the future will be performed. The integration of a mental health program specific for this group of infertile couples into the care program of this group can bring about significant result.

## Background

Infertility is one of the public health problems in the world that can threaten individual, marital, and social balance; because infertility is an unexpected tension and severe stigma for infertile couples [1] that can cause profound marital and emotional problems among them [2] and affect different aspects of people’s lives including their emotional and social performance [3]. Although assisted reproductive treatments have increased successfully the treatment of these couples, the use of these methods is costly and time-consuming and they should be repeated to achieve success that doubles the problems of these couples [4]. Among different types of infertility that require the use of assisted reproductive techniques, there are special groups that, because of the lack of access to suitable oocyte with the ability of fertilizing, resort to oocyte donation. The number of volunteers using this method has increased in recent decades [5, 6]. In the United States, about 13,000 attempts are made each year for pregnancy using donated oocytes [7]. The use of oocyte donation has increased from 9261 cycles, i.e. 10.7% of total ART cases, in 2002 to 16,976 cycles, i.e. 12.28% of ART cases, in 2006 [8].

Despite the therapeutic benefits and successes, the use of donated oocyte can cause challenges and harms for couples. The health of the donor, the quality of the donated oocyte, the potential for transmission of inherited diseases through donation [9], religious, ethical, legal and cultural criteria [10], and lack of genetic linkage of the succeeding generation with the mother [11] are among the important challenges in using this type of treatment. Additionally, social factors, including the relationship between families and the child resulting from donation, the time and how to inform the child, family, and relatives about the child’s genetic origins [12] lead to high levels of stress and tension for donated oocyte users [13]. Using a third party in fertility, particularly the oocytes of another person, may lead to more psychological reactions, especially in the part of the acceptor [14, 15].

Such challenges in this treatment method can differentiate the consequences of infertility in these couples from other infertile couples and consequently, create different needs including different psychological needs in these couples. Accordingly, couples using donated oocytes often expect to receive comprehensive and high quality care and treatment, and special attention should be paid to their mental health. The reason is that discovering the needs and priorities of clients in the infertility process [16, 17] and considering all aspects of their health, including mental health, are prerequisites of providing high-quality cares.

Mental health, as one of the most important aspects of health, can affect other aspects of one’s life including quality of life [18]. Accordingly, the health systems of countries seek to improve mental health in the society [19].

Despite the need to provide these infertile couples with mental health services, no special mental health program has been developed for these couples. Therefore, this study aims to develop a program based on the specific needs of couples using donated oocyte.

## Methods

This multi-level research, approved by the Ethics Committee of Isfahan University of Medical Sciences in Isfahan, Iran (IR.MUI.RESEARCH.REC.1398.312) will be conducted to develop a program for promotion of the mental health of infertile couples undergoing assisted reproductive treatment using donated oocyte. Informed consent will be obtained from all participants at all phases of the research. The development of this program will be based on the Talbot and Verrinder model, and include the following steps: determination of a specific topic for planning, initial design of the program, finalization of the program (using the views of experts in this area), implementation of the program, monitoring of the implementation of the program and evaluation of the program [20].

This research will be performed in three phases. In the first phase, the psychological needs of the couples will be identified using an exploratory mixed method. In the second phase, the program will be designed and validated based on the results of the first phase. In the third phase, a pilot study is designed to evaluate the impact of the program and its future implementation.

### Phase I: identification and evaluation of the psychological needs

This phase consists of a two-stage exploratory mixed study (qualitative-quantitative), the first stage of which covers Talbot program to determine the specific topic for planning. In the qualitative part, the psychological needs of the couples undergoing assisted reproductive method of oocyte donation are extracted. In addition, at this stage, the items of the tool for measuring these needs will be formulated based on the findings of the qualitative study. Then, using a cross-sectional study on the target population, the psychometric steps of the tool will be done and the level of psychological needs is also assessed.

### Qualitative study

In the qualitative study, the psychological needs of these couples are identified by conducting unstructured interviews and audio recordings. Purposive sampling method will be used at this stage. Iranian infertile couples undergoing assisted reproductive treatment using oocytes donation referring to infertility centers in Isfahan, Iran as well as health care providers will be used for gaining more comprehensive information. The aim of the study, the reason for the recording of the interview, the voluntary participation, the confidentiality of the information and the identity of the interviewee will be explained.

Inclusion criteria for couples using oocyte donation will be the ability to communicate appropriately, the ability to provide rich and complete information, and not suffering from any severe and persistent mental illness according to a psychiatrist and case documentation. Inclusion criteria for the personnel of the infertility centers will be willingness to participate in interviews. The unwillingness to continue their cooperation at any stage of the research and for any reason is considered as the exclusion criterion for each sample. At this stage, data are collected through open and unstructured interviews as well as on-site note taking. Informed consent will be obtained from the participants for recording the interviews, and the time, length and location of the interview will be chosen based on the participant’s preference. Interviews will be analyzed using the content analysis method (Granheim-Lundman method). Furthermore, based on the results of this stage, the items for measuring the psychological needs of the couple (PNS) will be designed.

To assure accuracy and reliability of the data, the four criteria of credibility, dependability, confirmability, and transferability will be used.

PNS questionnaire will be a self-report questionnaire with a five-point Likert scale (0–4) that is psychometrically assessed according to standard procedures.

The face validity of the questionnaire will be measured both qualitatively and quantitatively. To evaluate the face validity of the questionnaire qualitatively, all items will be evaluated for clarity, understanding, and simplicity by a number of members of the target group and providers infertility services to them, as well as a five-member team consisting of a psychiatrist, psychiatric nurse, and reproductive health specialist. The face validity will also be measured quantitatively by using the quantitative method of item impact. To this end, a 5-point Likert tool will be used in which the scores are assigned as “quite important” (5 points), “somewhat important” (score 4), “moderately important” (score 3), “slightly important” (score 2) and “not important” (score 1). Any item scored greater than 1.5 will be a good item and will be retained for later analysis.

To determine content validity qualitatively, the opinions of the experts (including physicians, psychiatrists, nurses, psychiatric nurses, and reproductive health professionals, midwives of infertility centers, psychologists, and tool design specialists) will be used. Content validity ratio (CVR) and content validity index (CVI) will be used to evaluate content validity quantitatively.

To determine the content validity ratio (CVR), each of the items of the questionnaire is judged by the experts in terms of necessity. The researcher will give the questionnaire to the experts and ask them to judge the items using the three-part scale of 1) “the item is necessary,” 2) the item is useful but not necessary,“ and 3) the item is not necessary”. The content validity ratio is then calculated using Lawshe formula. Based on Lawshe table, if the number of specialists is 10, the minimum acceptable value for CVR is 0.62. Accordingly, items whose CVR is above 0.62, will be retained [21]. To measure content validity ratio (CVR), the experts are asked to rate items using a 4-point Likert scale of 1) “not relevant”, 2) “relatively relevant”, 3) “relevant”, and 4) “quite relevant”.

### Quantitative study

The quantitative phase of this study is a cross-sectional study conducted on a number of infertile couples undergoing assisted reproductive treatment of oocyte donation. The sample size will be determined using the law of the ratio of the items to the respondents that equals 3 to 4 subjects per item and can be up to 10 subjects per item [22]. Inclusion criteria in the quantitative phase: the ability to read and write and no serious and persistent mental illness according to a psychiatrist.

Exploratory factor analysis is used to determine the construct validity using SPSS18 software. Moreover, confirmatory factor analysis will be performed using the AMOS18 web software.

For the evaluation of criterion validity, the General Health Questionnaire-28 (GHQ-28) introduced by Goldberg and Hillier will be used [23]. This questionnaire contains 28 four-choice questions to be answered by the subject. The GHQ-28 consists of four subscales of physical symptoms, anxiety and insomnia, social dysfunction and depression. Each subscale includes seven questions. The Likert system is used in scoring this questionnaire, that is, a score is given to each of the four choices (0, 1, 2, 3). The higher the score of the test, the lower will be the general health of the subject.

To determine internal consistency in this study, Cronbach’s alpha coefficient will be used for the whole tool, and factor analysis with the same samples of factor analysis stage for each factor separately. The stability in this study will be evaluated using a test re-test method on 10% of the participants of the cross-sectional study. Then, the score of the validated questionnaire and the criterion validity score are compared with the general health questionnaire and, finally, the psychological needs of these couples are determined using the developed tool.

### Phase II: designing a mental health program

In order to initialize and finalize the program (the second and third steps of Talbot program), an initial draft of the mental health program is prepared using the findings of the previous phase. Overall goals, specific goals, suggested strategies, and activities required for the mental health of couples undergoing oocyte donation will be developed. The focus of the program will be on the factors identified in the first phase of the research as the most important factors related to mental health. The following features will be considered in the design of the program:

- Development and evaluation of a program to address defined mental health problems and improve mental health in this specific group.
- Design a program appropriate for the specific needs of these clients
- Design a program with the ability to solve mental health problems in the simplest and most effective way.
- Accordingly, the action plans of the program will be designed using appropriate models.

Validation and finalization of the program is done using classic Delphi technique in four rounds and according to the priorities and goals of the program. The members will be used purposive sampling method. The panel members will consist of 10 experts (including physicians and psychiatrists, nurses and psychiatrist nurses, reproductive health professionals, midwives of infertility centers, and psychologists). Inclusion criteria of the panel participants: their scientific knowledge in the area of research and having at least 2 years of care providing in ART center. Exclusion criteria: their unwillingness to continue their cooperation in the research.

In the first round, the initial version of the program is prepared and, along with open-ended questions, will be presented to experts, and they are asked to comment on issues such as ease of implementation, cost effectiveness and the time required for implementation. After collecting the returned copies, they are organized and the structured questionnaire is used as the tool of the second-round. In the second round, the members are asked to rate and quantify the program items using a Likert scale. The analysis is then performed as a content analysis and, applying the comments of the experts, the revised version together with the evaluation checklist is provided to the experts, and then the revised program (the third version) is given to them. The strengths and weaknesses of the program are identified in this round, and the comments will be revised if needed. In the fourth round of the program, rankings, minority opinions and consensus items will be distributed among the panelists and the final program will be approved.

### Phase III

The fourth, fifth and sixth steps of the Talbot model are implementation, Monitoring of the implementation and evaluation of the program will be supervised. Owing to budget constraints, the program will not enter the pilot phase, but will be provided to the Isfahan Infertility Center for pilot, implementation and impact assessment.

## Discussion

The aim of this study is to develop a mental health promotion program for couples using donated oocytes. According to a logical premise, people are the best source for describing their own situation, needs, feelings, and experiences by their own words [24] Therefore, in the development of the present study protocol, the program is attempted to be developed based on the needs of the target population. Studies have shown that among needs, emotional and psychological needs are the most important ones [25–27]. According to the evidence, infertility-related psychological problems, including stress and anxiety, can be a contributing factor to the exacerbation of infertility and failure in the process of its treatment [28]. The importance of addressing the psychological and emotional needs of infertile couples is such that lack of attention to them can even play a role in influencing the outcomes of some infertility treatments [29] and, consequently, affect their mental health. The integration of a mental health program specific for this group of infertile couples into the care program of this group can bring about significant results. This program is based on the psychological needs of Iranian infertile couples. Therefore, considering the cultural structure of Iran, the program can be used in similar cultural structures. Additionally, given that Iran is the only country in the region that provides ART services using oocyte donation, the positive effects of this program can be extended to non-Iranian couples who use this technique in Iran.

However, differences in the disparate socio-cultural context of other countries may limit the application of this program elsewhere. Furthermore, lack of evaluation of the impact of the program on couples’ mental health, because of the project’s financial constraints, is one of the limitations of this study.

## Data Availability

Not applicable.

## Abbreviations

ART: Assisted Reproductive Techniques
CVI: Content Validity Index
CVR: Content Validity Ratio
GHQ-28: General Health Questionnaire-28
PNS: Psychological Needs Scale
